# Differential Expression of Kisspeptin System and Kisspeptin Receptor Trafficking during Spermatozoa Transit in the Epididymis

**DOI:** 10.3390/genes13020295

**Published:** 2022-02-02

**Authors:** Elena Mele, Raffaella D’Auria, Marika Scafuro, Marianna Marino, Silvia Fasano, Andrea Viggiano, Riccardo Pierantoni, Antonietta Santoro, Rosaria Meccariello

**Affiliations:** 1 Department of Movement Sciences and Wellness, University of Naples Parthenope, Via Medina 40, 80133 Naples, Italy; elena.mele@collaboratore.uniparthenope.it; 2 Department of Medicine, Surgery and Dentistry “Scuola Medica Salernitana”, Via S. Allende, 84081 Baronissi, Italy; radauria@unisa.it (R.D.); mamarino@unisa.it (M.M.); aviggiano@unisa.it (A.V.); 3 Department of Precision Medicine, University of Campania “Luigi Vanvitelli”, Via Costantinopoli 16, 80138 Naples, Italy; marika.scafuro@unicampania.it; 4 Department of Experimental Medicine, University of Campania “Luigi Vanvitelli”, Via Costantinpoli 16, 80138 Naples, Italy; silvia.fasano@unicampania.it (S.F.); riccardo.pierantoni@unicampania.it (R.P.)

**Keywords:** Kiss1, Kiss1R, spermatogenesis, testis, epididymis, spermatozoa

## Abstract

The hypothalamus–pituitary–testis axis controls the production of spermatozoa, and the kisspeptin system, comprising Kiss1 and Kiss1 receptor (Kiss1R), is the main central gatekeeper. The activity of the kisspeptin system also occurs in testis and spermatozoa, but currently the need of peripheral kisspeptin to produce gametes is not fully understood. Hence, we characterized kisspeptin system in rat spermatozoa and epididymis caput and cauda and analyzed the possible presence of Kiss1 in the epididymal fluid. The presence of Kiss1 and Kiss1R in spermatozoa collected from epididymis caput and cauda was evaluated by Western blot; significant high Kiss1 levels in the caput (*p* < 0.001 vs. cauda) and constant levels of Kiss1R proteins were observed. Immunofluorescence analysis revealed that the localization of Kiss1R in sperm head shifts from the posterior region in the epididymis caput to perforatorium in the epididymis cauda. In spermatozoa-free epididymis, Western blot revealed higher expression of Kiss1 and Kiss1R in caput (*p* < 0.05 vs. cauda). Moreover, immunohistochemistry revealed that Kiss1 and Kiss1R proteins were mainly localized in the secretory epithelial cell types and in contractile myoid cells, respectively. Finally, both dot blot and Elisa revealed the presence of Kiss1 in the epididymal fluid collected from epididymis cauda and caput, indicating that rat epididymis and spermatozoa possess a complete kisspeptin system. In conclusion, we reported for the first time in rodents Kiss1R trafficking in spermatozoa during the epididymis transit and Kiss1 measure in the epididymal fluid, thus suggesting a possible role for the system in spermatozoa maturation and storage within the epididymis.

## 1. Introduction

Spermatogenesis, the process of male gamete production, requires the occurrence of mitosis, meiosis, and differentiation of haploid round spermatids into spermatozoa [1]. Centrally and peripherally produced modulators direct spermatogenesis via endocrine, paracrine, and autocrine routes; in this respect, the hypothalamic gonadotropin releasing hormone (GnRH), pituitary gonadotropins, and gonadal sex steroids are the main actors in spermatogenesis onset, progression, and maintenance [1].

In the last years, the kisspeptin system has been included among the main central modulators of reproduction in mammalian species due to its ability to regulate upstream activity of GnRH secreting neurons. The kisspeptin system comprises the cleavage product of the Kiss1 precursor (i.e. kisspeptin (Kp)-54, originally known as metastin; Kp-14; Kp-13; and Kp-10] and the membrane G protein-coupled receptor, Kiss1R, previously known as Gpr54 [2]. All Kiss1 derived peptides share the C-terminal amino acid and have the ability to bind and activate Kiss1R. Natural or induced loss/gain of function due to mutations in *Kiss1*/*Kiss1r* genes cause hypogonadotropic hypogonadism and precocious puberty, respectively [3,4,5,6,7,8]; this system is also involved in the sex steroid feedback mechanisms [9] and in the epigenetic modulation of reproduction [10,11,12,13].

Apart from the brain, kisspeptin system is widely distributed in peripheral tissues. In males, it is highly expressed in the testis [14,15,16] and in the reproductive tract [17,18,19], raising the possibility that peripheral kisspeptin activity may directly sustain the formation and the physiology of spermatozoa. Accordingly, the selective reintroduction of *Kiss1R* in the hypothalamic GnRH cells of *Kiss1R* null mice rescues the basal activity of the reproductive axis, but it is not sufficient to completely restore spermatogenesis [20]. Furthermore, in vitro cell models [18,21,22], ex vivo treatments [23,24,25,26], testis explants [26], and in vitro co-colture models [27] have been used to investigate the involvement of the kisspeptin system in male reproduction, revealing a possible physiological role in spermatogenesis progression as well as steroid biosynthesis in several, but not all, of the species tested [15,16] for recent review. In fact, species-specific differences in the expression or localization of Kiss1/Kiss1R were reported in the testis of mammalian and non-mammalian vertebrates [16], and Kiss1R was detected in the spermatozoa of anuran amphibians, rodents, buffalos, dogs, and humans [17,25,28,29,30]. Kiss1 was recently detected in human seminal plasma with positive association to semen quality parameters [31].

Despite the fact that spermatozoa express Kiss1R, the role of kisspeptin signaling on male gametes and its functional role in fertilization is still poorly understood, and only few studies are available in the field. In fact, Kp-13 and Kp-10 affect intracellular Ca^2+^ mobilization in mice and humans, respectively [17,29], with consequences on in vitro fertilization ability in mice [17]. Effects on progressive sperm motility and transient sperm hyperactivation were reported in humans [29], but lack of any association between Kiss1R on sperm surface and the acquisition of sperm motility was reported in bulls [28]. 

The epididymis of mice [17], hamsters [32], and goats expresses both Kiss1 and Kiss1R [18]. Thus, the requirement of this system for sperm maturation was postulated. Accordingly, Kiss1R trafficking toward the plasma membrane during the transit from the epididymis caput to cauda was recently reported in dogs [30]; in parallel, kisspeptin immunoreactivity was demonstrated in the epididymal spermatozoa collection medium [30]. These findings revealed that the presence of the receptor on sperm surface matched the acquisition of the typical features of mature sperm within the epididymis and the possible need of kisspeptin signaling to gain sperm maturation and storage in the epididymis. 

Hence, in this manuscript, we fill the gap in the extra brain requirement of the kisspeptin system, characterizing and localizing Kiss1/Kiss1R in rat spermatozoa and epididymis caput and cauda. Then, we evaluated the possible presence of Kiss1 in the epididymal fluid.

## 2. Materials and Methods

### 2.1. Animals and Tissue Collection

Male Wistar rats aged 8–10 weeks (*n* = 10) were purchased from Harlan Laboratory (Harlan, Italy srl, Udine Italy) and maintained at standard temperature and humidity conditions with a 12 h light-dark cycle (lights on from 07:00 a.m. to 07:00 p.m.) and with free access to standard fresh food and tap water. Animals were sacrificed with anesthetic overdose (urethane). Blood was collected from left ventricle in EDTA treated tubes, centrifuged for 15 min at 800× *g* at 4 °C, and collected plasma was stored at −80 °C until used for Kiss1 detection assay.

Testis and epididymis were removed, and caput and cauda epididymis were dissected and cleared from tissue debris; then, collected tissues were rinsed with 1× Dulbecco Phosphate buffer (PBS). Five epididymis tracts from each animal were fixed in Bouin’s fluid, embedded in paraffin following standard procedures, and lastly, used for Harris hematoxylin and eosin (HE) staining and immunohistochemistry. Five more epididymis tracts were processed for the isolation and fixation of spermatozoa in 2% paraphormaldehyde, as reported in the next paragraph. The contralateral epididymis tracts and testis were frozen in liquid nitrogen and stored at −80 °C until processing for protein extraction and hormonal measurements, as reported in the next paragraph. 

All procedures were approved by local Ethical Committee of the University of Salerno and by the Ministry of Health, Directorate General of Veterinary Health and Food Safety (authorization number 66/2020 PR), and were conducted within the guidelines of the National Institutes of Health Guide for Care and Use of Laboratory Animals. 

### 2.2. Collection of Epididymal Spermatozoa, Epididymal Tissue and Fluid

Frozen caput and cauda epididymis (*n* = 10) were minced in 1 ml ice-cold PBS added to protease inhibitor cocktail (Santa Cruz, Biotechnology Inc. Dallas, TX, USA) and mixed at 4 °C on an orbital shaker for 5’ to allow spermatozoa to exit. After this step, caput and cauda epididymal pieces, diluted collection medium, and spermatozoa were processed as follows. Epididymis pieces were collected, transferred to new plates, and rinsed several times with 1 ml ice-cold PBS, until microscopic evaluation revealed the absence of spermatozoa in the washing buffer. Diluted epididymal fluid in collection medium was centrifuged at 800× *g* at 4 °C for 15 min to separate the spermatozoa-rich pellet, and then processed for ELISA assay or dot blot, as reported in the next paragraph. Spermatozoa rich pellets were resuspended in 1 ml PBS, filtered on gauge, three times resuspended, washed in ice-cold PBS, and centrifuged at 800× *g* at 4 °C for 15 min; the pellet was used for protein extraction.

For immunofluorescence analysis, caput and cauda spermatozoa were obtained from freshly collected epididymal tracts. To let the spermatozoa flow out from the ducts, caput and cauda of epididymis were separately immersed in PBS and cut into a few pieces. Spermatozoa were then filtered and fixed in 2% paraformaldehyde at a final concentration of 2 × 10^6^ cells/ml.

### 2.3. Total Protein Extraction 

Total protein extracts were prepared from rat testis (positive control), epididymis, and spermatozoa collected from caput and cauda epididymis [30] by using RIPA Buffer Lysis System (sc-24948, Santa Cruz Biotechnology, Inc. Dallas, TX, USA) following the manufacture’s instruction. In brief, tissues were homogenized in ice-cold RIPA buffer (1 g tissue/3 mL), supplied with a protease inhibitors cocktail (10 µL/mL RIPA), 100 mM sodium orthovanadate (10 µL/mL RIPA), and 200 mM phenylmethylsulfonyl fluoride (PMSF, 10 µL/mL RIPA). Spermatozoa were lysed by sonication in ice-cold RIPA buffer supplied with protease inhibitors, sodium orthovanadate, and PMSF. 

Lysates were incubated on ice for 30 min. Then, cleared protein extracts were collected by centrifugation at 11,000× *g* for 30 min at 4 °C. Lowry assay was used to measure protein concentration [33].

### 2.4. Western Blot 

Extracted proteins (30 µg) were analyzed by sodium dodecyl sulfate-polyacrylamide gel electrophoresis (SDS-PAGE), transferred to polyvinylidene fluoride (PVDF) membrane by TransBlot Turbo Transfer System (Bio-Rad, Milan, Italy), and processed for Western blot to assess the expression of Kiss1, Kiss1R, and MSJ-1, a specific marker of spermatozoa also known as DnaJB3 [34,35]; α-tubulin and α-actin were used as housekeeping genes. 

Western blot were carried out as previously reported [30] using the following primary antisera diluted in TBS 3% non-fat powdered milk solution overnight at 4 °C on an orbital shaker: goat polyclonal anti-Kiss1 raised against a peptide mapping at the C-terminus of Kiss1 of human origin (sc-18134, Santa Cruz Biotechnology,) diluted 1:1000; rabbit polyclonal anti-Kiss1R (sc-134499, Santa Cruz Biotechnology) diluted 1:1000; rabbit polyclonal anti MSJ-1 (Kind gift of Professor Giovanna Berruti, University of Milano Bicocca, Milan, Italy [34]) diluted 1:5000; monoclonal anti-α-tubulin (Elabscience E-AB-20036, USA) diluted 1:7500; and anti-α-actin polyclonal antibody (a-2066, Sigma-Aldrich, Milan, Italy) diluted 1:500 After washing three times in TBS/0.25% polyxyethylenesorbitan monolaurate (TBS-T)**,** membranes were then incubated in TBS 3% non-fat powdered milk solution for 1 h at room temperature (RT) with the corresponding secondary horseradish peroxidase conjugated anti-rabbit, anti-goat IgG (ImmunoReagents Inc., Raleigh, NC), or mIgGFc BP-HRP (sc-525409, Santa Cruz Biotechnology), respectively diluted 1:4000, 1:1000, and 1:500. After three washes in TBS-T, the immune complexes were detected by the Western blotting luminol reagent (sc-2048, Santa Cruz Biotechnology). For protein quantification by α-tubulin or α-actin, filters were first stripped at RT for 30 min in stripping buffer (sc-281698, Santa Cruz Biotechnology sc-281698), and then probed again with anti α-tubulin or anti α-actin antiserum.

### 2.5. Immunohistochemistry for Kiss1 and Kiss1R in Rat Seminiferous Tubules and Epididymis

Kiss1 and Kiss1R were localized in the corpus and cauda epididymis by immunohistochemistry. Bouin-fixed epididymis were paraffin-embedded following standard procedures. Deparaffinated sections (8 µm) were treated for 20 min with 0.3% H_2_O_2_ in methanol to inactivate endogenous peroxidase; then, slides were incubated in blocking solution (PBS 0.01 M pH 7.4, 1:30 mouse or rabbit serum according to primary antiserum) and 3% Bovine Serum Albumine (BSA) (Sigma-Aldrich) for 30 min at room temperature. Sections were incubated overnight in moist chamber at 4 °C with blocking solution containing primary antibody against Kiss1 (dilution 1:200, rabbit polyclonal anti-kisspeptin 10 raised against mouse Kp-10 (AB9754, Merk Life Science, Milan, Italy) [36]) or Kiss1R (dilution 1:100, sc-134499, Santa Cruz Biotechnology). Control sections were incubated with blocking solution alone (negative control). 

Then, the sections were washed twice in PBS 0.01 M pH 7.4 added with 0.3% Triton X-100 (5 min each), and once with PBS 0.01 M pH 7.4. Incubation with the corresponding IgG biotin conjugated secondary anti-sera (dilution 1:100, Santa Cruz Biotechnology) was carried out in blocking solution for 1 h at room temperature; thus, slides were incubated in Vectastain ABC reagents (Vector Laboratories, Inc., Burlingame, CA) for 1 h at room temperature. The immunoreaction signal was detected with 3,3-diaminobenzidine tetrahydrochloride (Sigma-Aldrich) in Tris-HCl 0.05 mM and 30% H_2_O_2_. Slides were observed under a light microscope (Leica 165 CTR500; Leica Microsystem) and images were captured using a high-resolution digital camera (Leica DC300F).

### 2.6. Immunofluorescence Analysis

Spermatozoa (0.5 × 10^6^) from caput and cauda were deposited on monolayer slides by Cytospin. Slides were washed three times with ice cold PBS for 10 min and blocked with a solution containing 10% BSA and 0.1% Triton X-100 in PBS for 1 h at RT. Then, cells were incubated overnight at 4 °C with primary antibodies: anti-kiss1R (1:200, BS-2501R, Bioss Antibodies, USA) and/or anti-MSJ-1 (1:200). Afterwards, slides were washed in ice cold PBS and incubated with the appropriate fluorescent-dye conjugated secondary antibodies (1:100 diluted, Santa Cruz Biotechnology) for 1 h at RT. Nuclei were stained with 4′,6-Diamidine-2′-phenylindole dihydrochloride solution (DAPI, 1.5 µg/ml).

Samples were then coverslipped with mounting gel (80% glycerol in PBS) and analyzed with an inverted Leica laser-scanning confocal microscope TCS SP5 (Leica Microsystems).

### 2.7. Dot Blot Analysis and Enzyme-Linked Immunosorbent Assay (ELISA)

After spermatozoa pull down, epididymal collection medium was cleared by centrifuging at 10,000× *g* at 4 °C for 15 min, used for dot blot or ELISA assay. The absence of spermatozoa or cell debris were evaluated under microscopy. For dot blot, the epididymal collection medium was processed, as recently reported [30]. Following the quantification by Lowry method [33], epididymal fluid proteins, BSA (negative control, Sigma-Aldrich), and rat testis proteins (positive control) (10 µg/sample) were applied by spotting directly on a methanol-activated PVDF membrane. The membrane was then processed, as previously described [30], for the detection of Kiss1 by means of incubation with the goat polyclonal anti-Kiss1 (sc-18134, Santa Cruz Biotechnology). Incubations with the aforementioned anti-MSJ-1 and anti-α-tubulin were also carried out to evaluate the possible contamination of protein from spermatozoa and epididymis cells. 

ELISA kit for rat Kiss1 (CEC559Ra, Cloud-Clone Corp., Katy, TX 77494, USA) was used for the quantification of Kiss1 in plasma and epididymal fluid (50 µL/sample, *n* = 4). Assay was run in duplicate following the manufacturer’s instructions. Intra-assay coefficient of variation: <10%; inter-assay coefficient of variation: <12%; detection range: 24.69–2000 pg/mL; minimal detectable Kiss1 dose: 9.22 pg/mL. 

### 2.8. Statistics 

Western blot signals were scanned, and protein levels were plotted, as quantitative densitometry analysis (optic density, OD) of signals. Values were expressed as protein of interest OD/housekeeping protein OD. Elisa test results were expressed as mean value ± standard deviation. ANOVA, followed by Student’s t-Test or Duncan’s Test, was carried out to assess the significance of differences (BioStat, AnalystSoft Inc., Walnut, CA). Differences were considered significant at *p* < 0.05. 

## 3. Results

### 3.1. Kiss1 and Kiss1R Protein in Spermatozoa 

Kiss1 and Kiss1R proteins were analyzed by Western blot in spermatozoa collected from caput and cauda epididymis. 

**Figure 1:**
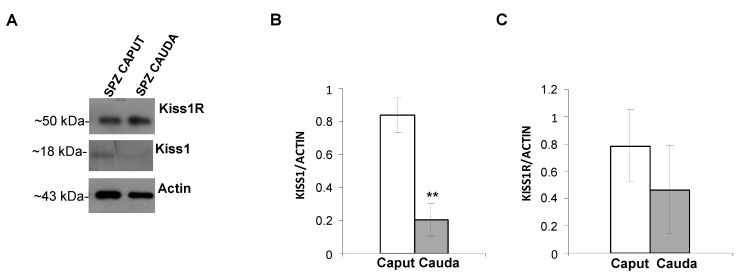
Expression of Kiss1 and Kiss1R in rat spermatozoa collected from epididymis caput and cauda. (**A**) Western blot for Kiss1 and Kiss1R. (**B**,**C**) Normalizations of Kiss1 precursor, Kiss1, and Kiss1R protein levels carried out against α-Actin. Asterisks indicate statistically significant differences (*p* < 0.001, *N* = 4). Asterisks indicate statistically significant differences (*p* < 0.01).

Rat spermatozoa expressed both Kiss1 and Kiss1R, but a different expression pattern was observed in caput and cauda epididymis (Figure 1A). In fact, Kiss1 signal was very faint in caput epididymis spermatozoa, and the detected protein levels significantly decreased in spermatozoa collected from cauda epididymis (*p* < 0.001) (Figure 1B). By contrast, Kiss1R protein was constantly expressed in the epididymis caput and cauda (*p* = 0.208) (Figure 1C).

**Figure 2:**
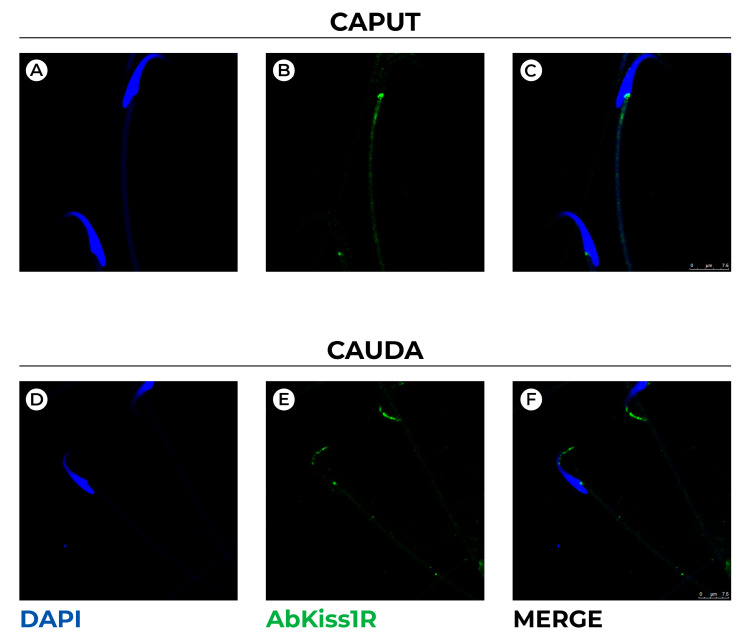
Confocal representative images showing immunofluorescence staining for Kiss1R (AbKiss1R, green) in rat spermatozoa collected from epididymis caput (**A**–**C**) and cauda (**D**,**E**). DAPI (blue) was used to counterstain sperm cell nuclei. Scale bar: 5 μm (**A**–**C**); 7.5 μm (**D**–**F**).

Hence, we used immunofluorescence detection to localize Kiss1R within rat spermatozoa collected from epididymis caput and cauda (Figure 2).

In spermatozoa collected from caput epididymis, Kiss1R was localized in the posterior region of sperm head marking the region that docks sperm tail to the elongated nucleus (Figure 2C). In spermatozoa collected from cauda (Figure 2D–F), epididymis Kiss1R was detected in the anterior part of sperm head. In detail, a scattered and pointed signal marks the ventral part of perinuclear theca, the apical hook shaped region of spermatozoa (i.e., the perforatorium), and ventrally, the nucleus follows the contour of plasma membrane.

**Figure 3:**
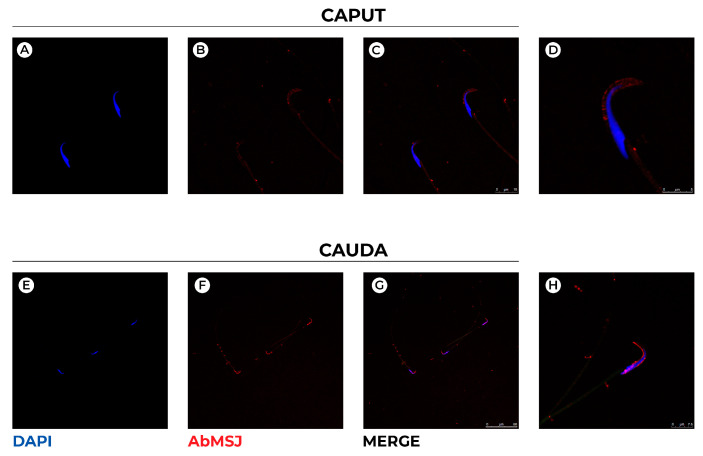
Confocal representative images showing immunofluorescence staining for MSJ-1 (AbMSJ, red) in rat spermatozoa collected from epididymis caput (**A**–**D**) and cauda (**D**–**H**). (**D**,**H**) represent higher magnification of spermatozoa showing MSJ-1 different distribution in caput and cauda epididymis. DAPI (blue) was used to counterstain sperm cell nuclei. Scale bar: 5 μm (**A**–**C**)**;** 7.5 μm (**D**–**F**).

Hence, double immunofluorescence analysis with the DnaJ protein, MSJ-1, that notably localizes at the outer surface of the acrosomal vesicle and the centriolar area [37] was carried out (Figure 3).

As expected, MSJ-1 marks the acrosomal vesicle and distributes preferentially in the dorsal outer acrosomal membrane of spermatozoa from caput epididymis (Figure 3C,D), whereas it extends both dorsally and ventrally in cauda spermatozoa (Figure 3F,H). The fluorescent intensity seems higher in cauda than in caput spermatozoa; immunoreactivity was also observed in centriolar area and the tail midpiece.

**Figure 4:**
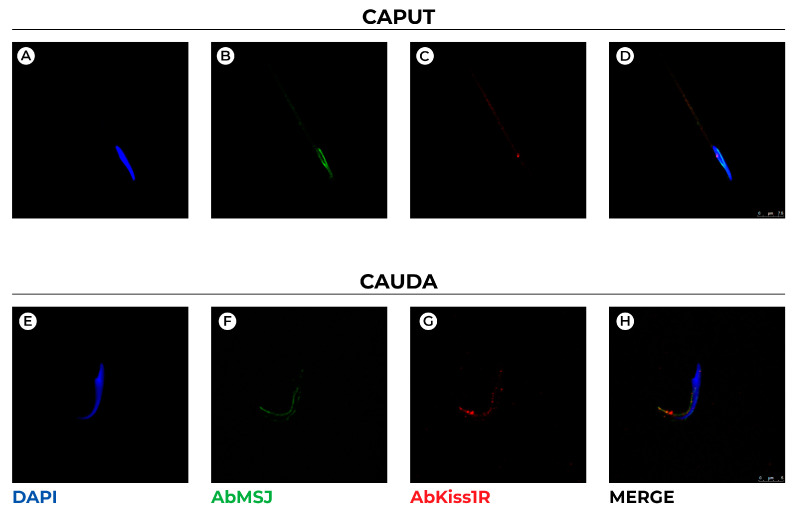
Confocal representative images showing double-immunofluorescence staining for MSJ-1 (AbMSJ, green) and Kiss1R (AbKiss1R, red) in rat spermatozoa collected from epididymis caput (**A**–**D**) and cauda (**E**–**H**). DAPI (blue) was used to counterstain sperm cell nuclei. Scale bar: 7.5 μm (**A**–**D**); 5 μm (**E**–**H**).

Double immunofluorescence for Kiss1R and MSJ-1 (Figure 4) corroborate previous localizations, and the different distribution of Kiss1R and MSJ-1 in caput and cauda spermatozoa also revealed a superimposed localization of both proteins in some spots of the huncus. Moreover, it appears that MSJ-1 surrounds Kiss1R distribution within the perforatorium and plasma membrane (Figure 4H).

### 3.2. Expression and Localization of Kiss1 and Kiss1R Protein in the Epididymis

**Figure 5:**
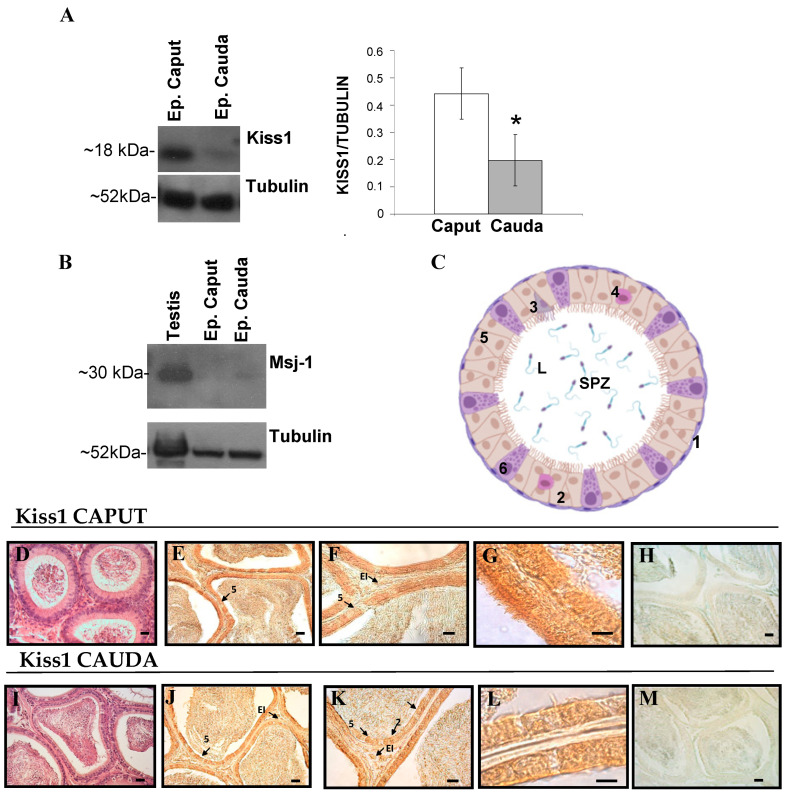
Expression and localization of Kiss1 in rat epididymis. (**A**) Western blot for Kiss1 in spermatozoa free epididymis caput and cauda. (**B**) Western blot for MSJ-1, used as a specific marker of spermatozoa. (**C**) Schematic representation of cell types in epididymal epithelium, with Kiss1 immunoreactive cells in beige. 1: Myoid cells; 2: basal cells; 3: apical cells, 4: Halo cell, 5: principal cells, 6: clear cells, L: lumen, SPZ: spermatozoa. (**D**–**H**) Histological sections of caput epididymis; (**I**–**M**) Histological sections of cauda epididymis. (**D**,**I**)**:** Haematoxylin-eosin staining. (**E**–**G**,**J**–**L**): Immunohistochemistry analysis for Kiss1. (**H**,**M**): controls of immunoreactions. Arrows indicate Kiss1 immunoreactive cells. Asterisk indicates statistically significant differences (*p* < 0.05).

**Figure 6:**
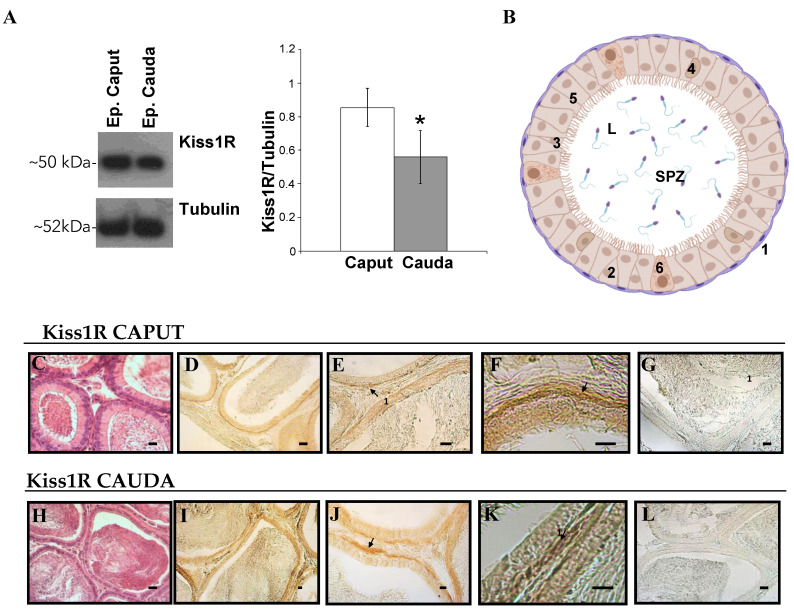
Expression and localization of Kiss1R in rat epididymis. (**A)** Western blot for Kiss1R in spermatozoa free epididymis (Ep.) caput and cauda. (**B**) Schematic representation of cell types in epididymal epithelium, with Kiss1R immunoreactive cells in violet. 1: Myoid cells; 2: basal cells; 3: apical cells, 4: Halo cell, 5: principal cells, 6: clear cells, L: lumen, SPZ: spermatozoa. (**C**–**G**) Histological sections of caput epididymis; (**H**–**L**) Histological sections of causa epididymis. (**C**,**H**) Haematoxylin-eosin staining. (**D**–**G**), (**I**–**L**): immunohistochemistry analysis for Kiss1R. (**G**,**L**): controls of immunoreactions. Arrows indicate Kiss1R immunoreactive cells. Asterisk indicates statistically significant differences (*p* < 0.05).

Western blot and immunohistochemistry were used to evaluate the expression and the localization of Kiss1 and Kiss1R within the epididymis (Figure 5 and Figure 6). High levels of Kiss1 protein were observed in caput epididymis (*p* < 0.05 vs. cauda) (Figure 5A). The possible contamination of spermatozoa in the protein extract was assayed through the analysis of MSJ-1 protein (Figure 5B). A very faint signal was detected for MSJ-1 in cauda epididymis only, with respect to testis used as positive control. 

Immunohistochemistry was used to analyze the distribution of Kiss1 within the epididymis. Kiss1 was localized in both caput and cauda epididymis within the epithelium, particularly in secretory principal cells and in basal cells; few elements in the epididymal interstitium were also immunostained (Figure 5E–G,J–L). Kiss1 immunostaining disappeared in control sections omitting primary or secondary antisera (Figure 5H,M). 

High levels of Kiss1R protein were observed in caput epididymis (*p* < 0.05 vs. cauda) (Figure 6A). In both caput and cauda Kiss1R, immunoreactivity was detectable in myoid cells all around the epididymal tubules (Figure 6D–F,I–K). 

Kiss1R immunostaining disappeared in control sections omitting primary or secondary antisera (Figure 6G,L). 

### 3.3. Dot Blot and ELISA

**Figure 7:**
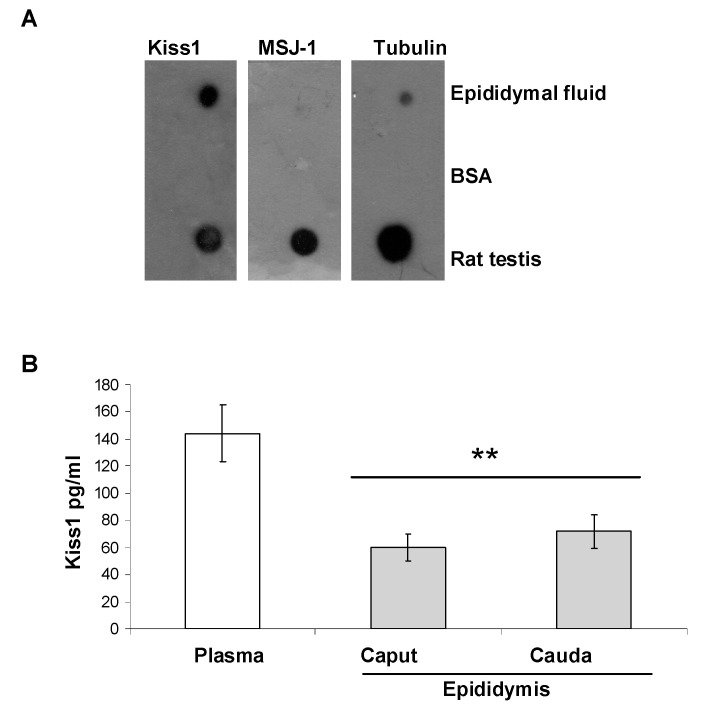
Kiss1 presence in the epididymal fluid. (**A**) Dot blot for Kiss1, MSJ-1, and α-tubulin carried out on epididymal fluid (10 μg proteins), Bovine Serum Albumine (BSA, negative control, 10 μg), and rat testis (positive control, 10 μg proteins). (**B**) Elisa assay for the measure of Kiss1 in plasma (positive control) and epididymal fluid collected from the epididymis caput and cauda of 8–10 weeks old rats (*n* = 4). Asterisks indicate statistically significant differences vs. plasma (*p* < 0.01).

Due to Kiss1 localization within the epididymis, the possible presence of Kiss1 in the epididymal fluid was evaluated. First, dot blot was carried out to assess the presence of Kiss1 immunoreactivity in the epididymal fluid in parallel to MSJ-1 and α-tubulin, used as markers of spermatozoa and epididymal cell lysis. Strong Kiss1 immunoreactive spots were observed in both epididymal fluids and testis (positive control), but not in BSA sample (negative control) (Figure 7A). MSJ-1 and α-tubulin spots were very faint compared to Kiss1 spot, resulting in a contamination of spermatozoa or epididymal cells smaller than 5%. 

Then, circulating Kiss1 levels were measured in plasma and in the epididymal collection medium by ELISA assay. High Kiss1 levels were detected in plasma (*p* < 0.001 vs. epididymis), and measurable Kiss1 levels were detected in caput and cauda epididymal collection medium (Figure 7B). Despite an increasing trend for Kiss1 level from caput to cauda, the difference was not statistically significant (*p* > 0.05). 

## 4. Discussion

Despite that the activity of kisspeptin system as gatekeeper of reproduction was recognized in the brain of mammalian and several non-mammalian species [2,9,38], possible kisspeptin activity outside the brain is not fully understood and remains debatable. Molecular and morpho-functional studies carried out in vertebrates [17,23,24,25,26,29,39,40,41,42,43,44] revealed that testicular kisspeptin may exert a role in the modulation of Leydig cell activity, germ cell progression, and sperm functions [8,16,19,25,27]. Indirect evidence that testicular kisspeptin may be secreted into peripheral seum was also shown in rodents [41]. 

In this manuscript, we report the presence of Kiss1 and Kiss1R at protein levels in rat spermatozoa collected from caput and cauda epididymis, and in epididymis itself. The epididymis is the anatomical structure that transports sperm from the rete testes to the vas deferens. This duct-like organ is functionally divided in segments characterized by their own specific microenvironment, in which cell to cell communications and functional exchanges of molecules like lipid mediators, proteins, and non-coding RNA within the epididymal-derived exosomes, called epididymosomes, occur [45,46]. The unique luminal environment of each epididymal region drives spermatozoa maturation. This process contributes to sperm motility and fertilizing competence, and is driven by enzymes and concentration gradients of signaling molecules that collectively induce substantial changes in sperm membrane composition, chromatin architecture, and migration of the cytoplasmic droplet from the proximal to caudal region of the tail. In a well-orchestrated sequence, the epididymis also concentrates and stores spermatozoa [46,47], and finally, shields and protects maturing spermatozoa from harmful substances through a blood–epididymis barrier and the release of antioxidant enzymatic defenses in the epididymal fluid [46]. 

Present data revealed the scanty presence of Kiss1 in spermatozoa collected from the epididymis caput, and an additional significant decrease in spermatozoa collected from epididymis cauda. Consistently, Hsu and coworkers (2014) [17] localized Kiss1 in the residual body of elongated spermatids and did not detect any Kiss1 immunofluorescent signals in spermatozoa collected from mouse epididymis cauda [17]. Thus, present data support the hypothesis that Kiss1 may be lost in mature spermatozoa. By contrast, Kiss1R was constantly expressed in spermatozoa collected from both caput and cauda epididymis. Furthermore, for the first time in rodents, our analysis revealed that the localization of Kiss1R in spermatozoa shifted the nucleus ventrally from the posterior head region to the anterior region during the transit of spermatozoa from epididymis caput to cauda. This may be due to intracellular trafficking occurring during the transit of spermatozoa in the different epididymal tracts. Alternatively, the deep biochemical remodeling of sperm membranes that is typical of sperm maturation may change the structure of the receptor, which appeared to be “masked” in caput epididymis and “unmasked” in cauda epididymis. Interestingly, in mature spermatozoa, the receptor was localized in the ventral area of the perinuclear theca and cell membrane and expanded until the most apical region of sperm head. The localization on the acrosome membrane was tested by co-localization assay with MSJ-1, a DnaJ protein that defines the cytoplasmic outline of the acrosome on dorsal and ventral portions [37]. Kiss1R only partially overlapped MSJ-1 in the huncus. To date, this is the first report in rodents analyzing the localization of Kiss1R in spermatozoa collected from both caput and cauda epididymis, since previous studies referred to cauda spermatozoa only [17]. However, present data differs in the localization on the dorsal region of the acrosomic membrane or equatorial segment and flagellar midpiece, reported in mice [17] and humans [29], respectively, but are consistent with recent evidence in dogs [30] that positively linked the trafficking of Kiss1R toward spermatozoa membrane during the epididymal sperm maturation [30].

The perinuclear theca is the major cytoskeletal–like element of sperm head that is underneath the acrosomic vesicle and surrounds the nucleus, with the exception of the tail implantation site (i.e. perifossal zone). In the hook shaped spermatozoa, the perinuclear theca comprises the subacrosomal layer, the post acrosomal sheet, and also the perforatorium, the curve pointed region particularly enlarged in falciform rat spermatozoa that encircles and extends beyond the nuclear apex. Its biogenesis involves the transport and deposition of proteins that, during the spermiogenesis, move ventrally from the microtubular manchette to reach the apical region of spermatid head [48]. In this respect, the intracellular trafficking of Kiss1R during sperm maturation recapitulates the trafficking occurring during sperm elongation. In mature spermatozoa, the post acrosomal sheet and perforatorium of the perinuclear theca represent a storage site for critical factors in early fertilization and zygotic events [48,49]. Consistently, the functional inhibition of Kiss1R by kisspeptin antagonist Kp-234 decreased the success of in vitro fertilization in mice [17]. Several aspects in spermatozoa physiology, like motility, capacitation, or acrosome reaction, depend on the mobilization of calcium from intracellular pools [50]. The few studies related to kisspeptin activity in sperm physiology revealed a role precisely in calcium mobilization [17]. In fact, in mouse spermatozoa, Kp-10 induced the mobilization of intracellular calcium, whereas, in humans, Kp-13 affects sperm progressive motility and transient sperm hyperactivation, but not acrosomal reaction [29]. Thus, Kiss1R trafficking occurring in spermatozoa head during sperm epididymal maturation may be functional for the acquisition of specific competence for fertilization. Eventually, for the first time in vertebrates, we measured significant levels of Kiss1 in the epididymal fluid, confirming the need of this system for sperm maturation and storage. 

At present, data related to Kiss1 and Kiss1R in epididymal cells are really poor and limited to Kiss1-precursor and Kiss1 protein localization in mice [17], Kiss1 and Kiss1R localization and mRNA levels at different developmental stages in goats [18], and *Kiss1/Kiss1R* mRNA in hamsters [32]. Interestingly, in the present manuscript, Western blot and immunohistochemistry also revealed the presence of Kiss1 and Kiss1R proteins in the epididymis, confirming data from mice and goats [17,18]. Nevertheless, both proteins were expressed at higher levels in caput epididymis, and the differential localization provided here opens new functional perspectives in the physiological activities of this system in rats. In fact, Kiss1 immunoreactivity was observed in the epithelial cells, particularly in the basal and secretory principal cells. The possible secretion of Kiss1 within the epididymal fluid was consistent with the decrease in the levels of Kiss1 protein in the cauda epididymis and was finally confirmed by the dot blot and the ELISA test. Differently to goats [18], Kiss1R was mainly localized within the contractile myoid cells which move sperm and fluids along the male reproductive tract. Hence, the presence of Kiss1 in the epididymal fluid (present data), and Kiss1/Kiss1R expression and localization within the epididymis strongly suggest the need of kisspeptin signaling as paracrine actor to move spermatozoa in the lumen from caput to cauda, where they are concentrated and stored until ejaculation. Consistently, in vitro, Kp-13 also modulates via Kiss1R, the contractions of circular and longitudinal colonic muscle promoting gastrointestinal motility [51], whereas, the anti-metastatic effects of kisspeptin just occur through the inhibition of cancer cells motility and invasion ability [52].

Present data confirms the presence of Kiss1 in the epididymal fluid, as recently suggested in dogs by dot blot assay [30]. Here, by ELISA assay for the first time in rodents, we measure detectable levels of Kiss1 in both caput and cauda epididymis, in parallel to plasma Kiss1. The possible contamination of epididymal fluid with spermatozoa or cell cytoplasm from broken epididymal cells, all expressing Kiss1, may represent a weakness in this study. However, the collected epididymal fluid was tested by dot-blot for both MSJ-1 (specific markers of spermatozoa), and tubulin was a widely expressed protein, revealing a contamination rate smaller than 5%. 

The main limitation of this study is the lack of functional data through the activation or impairment of Kiss1R on spermatozoa collected at different tracts of the epididymis in physiological conditions, but also in spermatozoa-defective animal models. Thus, additional studies are required in the field to make more stringent our observations, and to ascertain the functional role of the kisspeptin system in sperm physiology. However, apart from the centrally mediated positive effects on sperm quality, two previous studies only reported the direct effects of Kp-13 in human spermatozoa [29] and the consequences of Kiss1R antagonism on the fertilizing ability of mature mouse spermatozoa [17]. A recent study recently described the first association between Kiss1R on sperm surface and sperm maturation in dog model [30]. Due to its antioxidant properties [53,54], Kiss1 presence in the epididymal fluid, but also in seminal plasma, may represent a marker of sperm quality and a protective shield for spermatozoa. To the best of our knowledge, currently only one study investigated the possible role of kisspeptin in the seminal plasma. This study was carried out in a cohort of 666 healthy Chinese students, and first compared kisspeptin levels in seminal plasma to blood plasma, then associated kisspeptin levels in seminal plasma to sperm quality. Higher kisspeptin level in the seminal plasma than blood was found. Furthermore, a positive association between kisspeptin in seminal plasma and sperm quality (i.e., sperm concentration and total motile sperm count) was reported [31]. If additional studies in sub(in)fertile men confirm this association, the evaluation of kisspeptin in the seminal plasma could be used as marker of sperm quality. 

Taken together, present data may be relevant for the description of evolutionarily conserved processes, but also is promising and useful for further research on the involvement of kisspeptin in male fertility. 

## 5. Conclusions

In conclusion, we demonstrate the existence of a complete kisspeptin system in rat spermatozoa and epididymis. For the first time in rodents, we demonstrate the differential expression of the system in caput and cauda epididymis. Furthermore, we provide evidence of Kiss1 presence in the epididymal fluid and Kiss1R trafficking in spermatozoa head during the transit from epididymis caput to cauda, where mature spermatozoa are stored for ejaculation. Thus, a new evolutionarily conserved function for the kisspeptin system in sperm epididymal maturation and storage may be suggested. 

## Data Availability

Not applicable.
